# Single nucleotide polymorphism in the microRNA-199a binding site of HIF1A gene is associated with pancreatic ductal adenocarcinoma risk and worse clinical outcomes

**DOI:** 10.18632/oncotarget.7263

**Published:** 2016-02-08

**Authors:** Xiuchao Wang, He Ren, Tiansuo Zhao, Weidong Ma, Jie Dong, Shengjie Zhang, Wen Xin, Shengyu Yang, Li Jia, Jihui Hao

**Affiliations:** ^1^ Department of Pancreatic Cancer, Tianjin Medical University Cancer Institute and Hospital, Key Laboratory of Cancer Prevention and Therapy, National Clinical Research Center for Cancer, Tianjin 300060, China; ^2^ Department of Tumor Biology and Comprehensive Melanoma Research Center, H. Lee Moffitt Cancer Center & Research Institute, Tampa, FL 33612, USA; ^3^ Centre for Haemato-Oncology, Barts Cancer Institute, Queen Mary University of London, London EC1M 6BQ, United Kingdom

**Keywords:** hypoxia-inducible factor-1 alpha (HIF-1α), microRNA-199a (miR-199a), pancreatic ductal adenocarcinoma (PDAC), single nucleotide polymorphism (SNP)

## Abstract

Hypoxia-inducible factor-1 alpha (HIF-1α) is over-expressed in many cancers including pancreatic ductal adenocarcinoma (PDAC) and correlated with poor prognosis. We aim to determine the effect of germline genetic variants on the regulation of the homeostasis of the miRNA-gene regulatory loop in HIF1A gene and PDAC risk. HIF1A rs2057482 single nucleotide polymorphism (SNP) was genotyped in 410 PDAC cases and 490 healthy controls. The CC genotype SNP HIF1A is significantly correlated with PDAC risk (OR = 1.719, 95% CI: 1.293–2.286) and shorter overall survival (OS, P<0.0001) compared with the CT/TT alleles group. The C/T variants of rs2057482, a SNP located near the miR-199a binding site in HIF1A, could lead to differential regulation of HIF1A by miR-199a. Specifically, the C allele of rs2057482 weakened miR-199a–induced repression of HIF-1α expression on both mRNA and protein levels. In the PDAC tissue, individuals with the rs2057482-CC genotype expressed significantly higher levels of HIF-1α protein than those with the rs2057482-CT/TT genotype (P<0.0001). Both the CC genotype of SNP HIF1A and increased HIF-1α expression are significantly associated with shorter OS of patients with PDAC. After adjusted by TNM staging, differentiation grade, and the levels of CA19-9, both SNP HIF1A and HIF-1α expression retained highly significance on OS (P<0.0001). Taken together, our study demonstrates that host genetic variants could disturb the regulation of the miR-199a/HIF1A regulatory loop and alter PDAC risk and poor prognosis. In conclusion, the rs2057482-CC genotype increases the susceptibility to PDAC and associated with cancer progression.

## INTRODUCTION

Pancreatic ductal adenocarcinoma (PDAC) is the fourth highest cause of death from cancer in the western world [1], and is the fifth most common cause of cancer death in China [2]. The mortality rate of this malignancy is similar to its incidence rate and most of patients die within one year of diagnosis. The devastating feature of PDAC results mainly from the delay in diagnosis, the tendency of this cancer to rapidly metastasize to lymph nodes and distant organs, and its resistance to conventional therapies. Important progress has been made in the identification and characterization of molecules involved in the pathogenesis of PDAC in the past decade [3-8]. However, advances in its diagnosis, therapeutic intervention, and survival benefit are still poor. Therefore, there is a tremendous demand to have an improved understanding of the molecular mechanisms that underlie the aggressive behavior of this malignancy.

Hypoxia is a common feature of solid tumors as a consequence of poor tumor vascularization. The transcription factor hypoxia-inducible factor-1 (HIF-1) is a key regulator responsible for the induction of genes that facilitate adaptation and survival of tumor cells from hypoxic microenvironment and confer the tumor a worse malignant phenotype [6, 9-11]. As a heterodimeric complex, HIF-1 consists of a hypoxically inducible subunit HIF-1α and a constitutively expressed subunit HIF-1β. Overexpression of HIF-1α was found in various types of cancers of both human and mice and is an unfavorable prognostic factor in different cancers [12-15]. HIF-1α overexpression in human cancers is considered as consequences of intra-tumoral hypoxia as well as genetic alterations [16-18]. We have previously demonstrated that overexpression of HIF-1α is associated with advanced tumor stages in patients with PDAC [5, 6, 19-22] and proposed that HIF-1α is an attractive target for the development of anticancer agents [23-25]. However, the molecular mechanism of an increased constitutive expression of HIF-1α in PDAC is still elusive.

Genetic polymorphisms are responsible for inter-individual variation and diversity and have been considered as the main genetic elements involved in the development of common and complex diseases [26-28]. A number of single nucleotide polymorphisms (SNPs) associated with tumor progression have been identified in the HIF-1α gene [5, 29-32]. Several recent studies have further highlighted that the miRNA-related SNPs, especially those located within miRNA complementary sites, can remarkably alter the biogenesis and/or function of the corresponding miRNA [33-36]. Most miRNAs bind to target sequences located within the 3′-untranslated region (3′UTR) of mRNAs by base pairing, resulting in the cleavage of target mRNAs or repression of their translation [27, 37, 38]. Recent studies demonstrated that HIF-1α expression is regulated by miRNAs [39-41] and SNP rs2057482 in HIF1A gene is significantly associated with clinical outcomes of hepatocellular carcinoma patients after surgery [32]. We therefore hypothesize that polymorphism of HIF1A gene could lead to uncontrolled expression of HIF-1α protein in cancer.

To study the regulatory role of polymorphism of HIF1A gene in its protein expression, we first used a case-control study to demonstrate that the HIF1A SNP rs2057482 is an important genetic variant for PDAC risk and poor prognosis. We then further functionally validated the SNP rs2057482, which is located near the miR-199a seed-binding site in the 3′UTR of HIF1A. Finally, we performed a number of wet-laboratory experiments using reporter gene assays and validated that rs2057482 could lead to differential regulation of HIF-1α by miR-199a.

## RESULTS

### The characteristics of study subjects

We first used a population-based approach to evaluate the impact the HIF1A 3′UTR SNP (rs2057482) on clinical prognosis in 410 patients with PDAC, with 490 healthy donors as normal controls. Genotype frequencies among the controls did not show significant departures determined by Hardy–Weinberg equilibrium analysis (P=0.786). The patients and controls groups (Table 1) were comparable in terms of age and gender distribution. The average age at the diagnosis for PDAC patients was 60.5 ± 10.3 years, which was similar to those for the controls at recruitment (59.3 ± 11.1 years, P=0.113). PDAC patients showed significantly increased populations with histories of pancreatitis (P<0.001), type II diabetes (P<0.001) and smoked more than 20 pack-years (P<0.001) compared with the control group.

**Table 1:** The characteristics of study subjects

Characteristics	Patients	Volunteers	P^#^
Case number	410	490	
Age (years, mean ± SD)	60.5±10.3	59.3±11.1	0.113
Gender			
Men	259 (63.2%)	314 (64.1%)	0.777
Women	151 (36.8%)	176 (35.9%)	
Pack-year of smoking			<0.001
Never	266 (64.9%)	343 (70.0%)	
<20 pack-years	46 (11.2%)	90 (18.4)	
≥20 pack-years	98 (23.9%)	57 (11.6)	
History of pancreatitis			<0.001
Yes	20 (4.9%)	4 (0.8%)	
No	390 (95.1%)	486 (99.2%)	
History of type II diabetes			<0.001
Yes	119 (29.0%)	45 (9.2%)	
No	291(71.0%)	445 (90.8%)	

# Student's *t*-test for age distributions between patients and controls; and two-sided χ^2^ test for other selected variables between patients and controls.

### PDAC patients show an increased frequency of the C allele of the HIF1A gene SNP rs2057482

The genotype distributions and allele frequencies for HIF1A gene SNP rs2057482 were analyzed by DNA sequencing (Supplementary Figure 1) and statistically compared between PDAC patients and normal controls (Table 2). There was a significant difference in the genotypes of the HIF1A SNP rs2057482 between the PDAC patients and controls (P<0.05). Using the unconditional logistic regression analysis, we found that patients with the CC genotype showed a significantly higher risk of PDAC (OR=1.719, 95% CI: 1.293–2.286, P<0.05) compared with the T (TT+CT) allele carriers, indicating that the C allele SNP rs2057482 is associated with PDAC risk.

**Table 2:** rs2057482 SNPs in 410 PDAC patients and 490 healthy donors

Genotypes	Frequencies		*P*	ORs (95% CI)	*P*
	Patients	Volunteers			
CC	301 (73.4%)	302 (61.6%)	<0.001	2.626 (1.533-4.498)	<0.001
CT	69 (16.8%)	154 (31.4%)		1.180 (0.727-1.916)	
TT	40 (9.8%)	34 (6.9%)		1.000 (ref)	
CC	301 (73.4%)	302 (61.6%)	<0.05	1.719 (1.293-2.286)	<0.05
CT+TT	109 (26.6%)	188 (38.4%)		1.000 (ref)	
C allele	671 (81.8%)	758 (77.3%)	<0.05	1.319 (1.046-1.664)	<0.05
T allele	149 (18.2%)	222 (22.7%)		1	

Data were analyzed by logistic regression and expressed as ORs (95% Cl range) and *P* values.

### The C allele SNP of HIF1A rs2057482 is associated with poor clinical outcomes in PDAC patients

The prognostic impact of the SNPs (rs2057482) was evaluated by stratification of clinic-pathological characters of patients with PDAC (Table 3 and Supplementary Table 1). 269 cases of PDAC who had been followed up for at least 12 months (the median follow-up time for this cohort was 24 months) were selected for analysis the clinical outcomes. The HIF1A genotypes were significantly correlated with primary tumor size (T stage) (P=0.002) and lymph node metastasis (P=0.022). Patients with CC genotype showed larger tumor sizes and higher frequencies of lymph node metastasis (N) compared to those with other variant alleles (CT or TT genotypes). There is no significant association between SNP with serum CA19-9 levels, the clinical stages (TNM), or grades of differentiation, respectively.

**Table 3:** Relation of rs2057482 SNPs to clinical characteristics of 269 PDAC patients

Characteristics	rs2057482		*P*
	CC	CT/TT	
T (T1:T2:T3:T4)	4:24:100:74	8:17:23:19	0.002
LN metastasis (N0:N1)	91:111	41:26	0.022
Distant metastasis(M0:M1)	134:68	49:18	0.303
Clinical stage (I:II:III:IV)	15:63:56:68	9:21:19:18	0.429
Tumor grade (G1:G2:G3)	35:80:87	17:29:21	0.059
CA199 (normal: increased)	42:160	13:54	0.808
HIF-1α (L:M:H)	16:80:106	36:26:5	<0.001

Note: P values were calculated by the Spearman rank correlation test (n=269). T: Tumor size, T1: tumor limited to the pancreas, 2cm or less in greatest dimension. T2: tumor limited to the pancreas, more than 2cm in greatest dimension. T3: tumor extends beyond the pancreas but without involvement of the celiac axis or the superior mesenteric artery. T4, tumor involves the celiac axis or the superior mesenteric artery. LN, regional lymph node metastasis.

**Figure 1:**
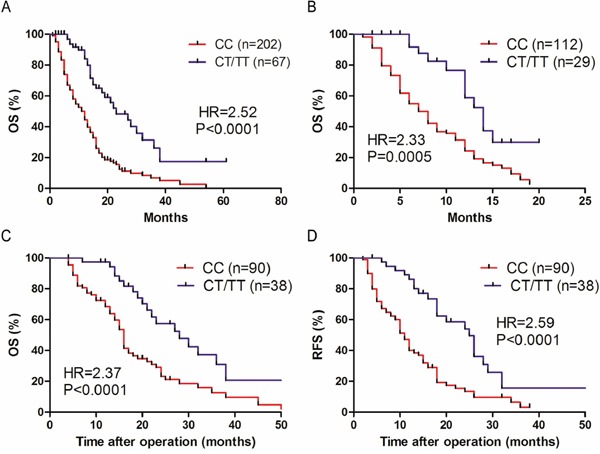
The association between SNP of HIF1A and survival rate in patients with PDAC **A.** Kaplan–Meier survival curves of overall survival (OS) of 269 PDAC patients with the CC genotype verses the CT/TT phenotypes. **B.** Kaplan–Meier curves of OS for 141 advanced PDAC patients with the CC genotype verses the CT/TT phenotypes. **C, D.** OS (C) and relapse-free survival (RFS) (D) of 128 PDAC patients who undergoing surgical resection, presented as CC verses CT/TT genotype groups. Data were analyzed by the log-rank test and the Kaplan–Meier curves were generated by using GraphPad Prism software.

We subsequently determined whether the rs2057482 genotypes affect overall survival (OS) of patients with PDAC. Kaplan–Meier survival curves showed the patients with CC genotype (n=202) is significantly associated with a shorter OS compared with the TT+CT genotypes (n=67) (HR=2.52; P<0.0001) (Figure 1A). The median OS of patients with CC genotype was 12 months verses 23 months of patients with other genotype SNPs. In patients with advanced PDAC, CC genotype (n=112) is also significantly associated with a shorter OS compared with the TT+CT genotypes (n=29) (HR=2.33; P=0.0005) (Figure 1B), with a median OS of 7 months verses those of 14 months. Similarly, the PDAC patients with CC genotype SNP showed shorter OS (HR=2.37; P<0.0001) and RFS (HR=2.59; P< 0.0001) compared with patients of TT+CT genotypes after surgical resection (Figure 1C and 1D). These results demonstrate that the CC genotype SNP of rs2057482 could predict the clinical outcome for PDAC patients before and after surgical resection.

### Allelic variants of rs2057482 alter the levels of HIF-1α mRNA and protein in PDAC tissues

**Figure 2:**
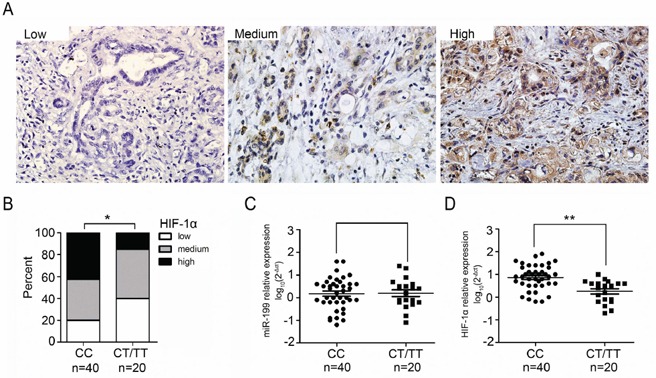
Relative expression levels of miR-199a and HIF-1α in PDAC tissues **A.** Immunohistochemical staining of HIF-1α in PDAC with low-, medium- and high-levels of HIF-1α expression; **B.** The frequency distribution of levels of HIF-1α expression between CC and CT/TT genotype groups (n = 60, *P < 0.05); **C.** Levels of miR-199a expression in PDAC tissues of CC or CT/TT genotype groups (n=60); **D.** Comparison of HIF-1α protein expression in samples with the CC genotype and CT/TT genotypes (n = 60, **P< 0.01).

Expression of HIF-1α protein was determined in the tissue specimens from 269 PDAC patients and observed a heterogeneous expression pattern of HIF-1α proteins among these samples (Figure 2A). Higher expression of HIF-1α is significantly associated with shorter OS and RFS (P<0.0001 with HR=6.20 to 4.01 (Supplementary Figure 2). We designed a semi-quantitative method based on the staining intensity and frequency of HIF-1α positive cells. The C variant of rs2057482 was significantly correlated with higher levels of HIF-1α expression (Table 3, Supplementary Figure 3). We randomly selected 60 PDAC patients, 40 patients with CC genotypes and 20 patients with CT/TT genotypes. Expression of HIF-1α protein, HIF-1α mRNA, and miR-199a were determined in the epithelial cells of the tumor tissues. The levels of HIF-1α protein were significantly higher in the PDAC tissue with the CC genotype than those with the CT/TT genotypes (P<0.05) (Figure 2B). The miR-199a expression showed no difference among these genotypes (P>0.05) (Figure 2C). Furthermore, there was significantly increased expression of HIF-1α mRNA in the PDAC tissue of individuals with the CC genotype compared to those with the CT/TT genotypes (P<0.01) (Figure 2D). These results demonstrate that the CC allele SNP of HIF1A gene is, at least partly, associated with an increased expression of HIF-1α, both RNA and protein.

### Both CC genotype HIF1A SNPs and higher HIF-1α protein expression are associated with adverse prognosis

Univariate and multivaritate Cox regression analyses were used to further determine the association between HIF1A SNPs or HIF-1α protein expression with clinical outcomes of patients with PDAC and compared with standard prognostic markers. All clinical features, HIF1A SNPs, and HIF-1α protein levels were analyzed with the univariate Cox regression first (Table 4). TNM (P<0.0001), tumor size (P=0.001), lymph nodes metastasis (P<0.0001), CA19-9 (P=0.005), grade of differentiation (P<0.0001), HIF1A SNPs (P<0.0001), or HIF-1α expression (P<0.0001) all showed significant association with OS of patients with PDAC but age has no impact of PDAC prognosis. Using multivariate analysis with adjustment by all clinical prognostic markers, HIF1A SNPs (P=0.001) and HIF-1α expression (P=0.001) were retained their significant prognostic power. After backward stepwise selection, TNM (P<0.0001), differentiation grade (P<0.0001), CA19-9 (P=0.026), HIF1A SNPs (P<0.0001) and HIF-1α expression (P<0.0001) were retained significance for predicting OS in the final model of the multivariate analysis (Table 4). These results indicate that both HIF1A SNPs and HIF-1α expression may be independent prognostic markers for OS of patients with PDAC.

**Table 4:** Uni- and multivariate analysis for covariables of PDAC patients

Covariables	Overall survival		Overall survival		Overall survival	
	HR (95% CI)	*P*	HR (95% CI)	*P*	HR (95% CI)	*P*
	Univariate		Multivariate^(1)^		Multivariate^(2)^	
**HIF1A SNP**	2.87 (1.93-4.26)	<0.0001	2.18 (1.35-3.05)	0.001	2.34 (1.47-3.71)	<0.0001
**HIF-1α^LOW^** **HIF-1α^MEDIA^** **HIF-1α^HIGH^**	Ref. 1.78 (1.11-2.85) 6.21 (3.91-9.88)	<0.0001 0.016 <0.0001	1.23 (0.69-1.91) 2.22 (1.25-3.94)	0.001 0.651 0.006	1.08 (0.64-1.81) 2.22 (1.26-3.89)	<0.0001 0.770 0.006
**TNM I** **TNM II** **TNM III** **TNM IV**	Ref. 0.94 (0.52-1.71) 2.38 (1.29-4.36) 4.93 (2.68-9.09)	<0.0001 0.846 0.005 <0.0001	Ref. 0.85 (0.34-2.12) 1.37 (0.46-4.09) 4.13 (1.58-10.79)	<0.0001 0.726 0.568 0.004	Ref. 1.13 (0.60-2.10) 2.64 (1.39-5.05) 5.91 (3.03-11.56)	<0.0001 0.710 0.003 <0.0001
**Grade 1** **Grade 2** **Grade 3**	Ref. 1.59 (1.03-2.47) 3.43 (2.22-5.29)	<0.0001 0.036 <0.0001	Ref. 1.84 (1.16-2.92) 3.45 (2.14-5.56)	<0.0001 0.010 <0.0001	Ref. 1.88 (1.19-2.97) 3.31 (2.09-5.26)	<0.0001 0.006 <0.0001
**CA199**	1.71 (1.17-2.49)	0.005	1.78 (1.15-2.74)	0.010	1.62 (1.06-2.48)	0.026
**T1** **T2** **T3** **T4**	Ref. 2.12 (0.80-5.58) 2.43 (0.98-6.02) 4.04 (1.62-10.09)	0.001 0.129 0.055 0.003	Ref. 2.09 (0.75-5.84) 2.33 (0.77-7.06) 3.20 (0.95-10.73)	0.303 0.161 0.136 0.060		
**Metastasis**	1.99 (1.48-2.68)	<0.0001	1.02 (0.69-1.52)	0.910		
**Age**	1.01 (0.99-1.02)	0.241	1.01 (0.99-1.03)	0.224		

Note: Data shown were analyzed by Cox proportional hazard model (n=269). Abbreviations: HR, hazard ratio; 95% CI, 95% confidence interval; *P*, P value; Multivariate^(1)^: the full model, all variables are included; Multivariate^(2)^: final variables after the backward stepwise selection; HIF1A SNP: set up as CC alleles verses CT/TT genotypes; HIF-1α: indicates levels of HIF-1α protein expression; tumor size: T, T1: tumor limited to the pancreas, 2cm or less in greatest dimension. T2: tumor limited to the pancreas, more than 2cm in greatest dimension. T3: tumor extends beyond the pancreas but without involvement of the celiac axis or the superior mesenteric artery. T4, tumor involves the celiac axis or the superior mesenteric artery; metastasis: lymph node metastasis; CA199: >39 verses <39.

The Spearman's correlation analysis (Table 3 and Supplementary Table 1) showed that HIF1A SNPs and HIF-1α express have strong correlation between each other (P<0.001). To further evaluate the clinical causal relationship between HIF1A SNPs and HIF-1α expression, the effect of HIF-1α expression on patients OS were either stratified or adjusted by HIF1A SNPs and analyzed by multivariate Cox regression analysis (Supplementary Table 2). The impact of HIF-1α on OS still retains highly significant (P<0.0001). This suggests that the constitutive expression of HIF-1α is regulated by HIF1A SNPs.

### SNP rs2057482 C→T near the miR-199a binding site in the HIF1A 3′UTR is a functional regulatory site

**Figure 3:**
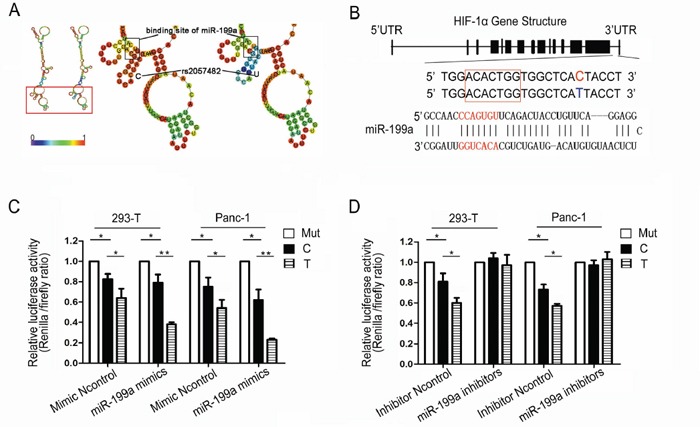
miR-199a differential regulation of the SNP rs2057482 **A.** The predicted secondary structure of the HIF-1α mRNA. The secondary structures of the 3′UTR of HIF1A were predicted by inputting two 302-nt long DNA sequences centering rs2057482 into RNA fold, with either the rs2057482-C (left) or rs2057482-T (right) allele. The figures and the values of minimum free energy (MFE) were generated by RNAfold (http://rna.tbi.univie.ac.at); **B.** Schematic representation of the sequences of human miR-199a and its target site the 3′ UTR of HIF1A. The rs2057482 SNP is located 8 nucleotides (nt) downstream of the “seed complementary sequence” of miR-199a in HIF1A. **C, D.** Effects of rs2057482 genotypes on the expression of HIF1A gene in HEK-293T and Panc-1 cell lines. Forty-eight hours after transfection of the reporter gene and miR-199a mimic, the roles of construct with C or T allele on relative luciferase activity were compared in HEK-293T and Panc-1 cell lines (*P <0.05, **P <0.01). Data shown are mean ± SD of three independent experiments.

We next examined whether genetic variants of rs2057482 could alter the local secondary structure of the HIF-1α mRNA based on the minimum free energy (MFE) method. RNAfold, online RNA secondary structure prediction software, predicted that allelic variants of rs2057482 are able to alter the local mRNA secondary structure including that of the miR-199a binding site. The minimum free energy changed from −61.9kcal/mmol to −59.3kcal/mmol when the nucleotide at the rs2057482 locus changed from C to U (Figure 3A). By comparison of the genomic location of rs2057482 with the binding site of miR-199a, we found that rs2057482 was located 8 bp downstream of the binding site of miR-199a in the 3′UTR of the HIF-1α mRNA (Figure 3B). We then performed a reporter gene assay to validate the computational prediction and test the hypothesis that miR-199a more robustly regulates the T allele. We cloned the HIF1A 3′UTR fragments into a luciferase reporter vector. Reporter gene vectors containing either the T or C allele (or the mutant of the miR-199a binding site in HIF1A 3′UTR), the miR-199a mimic (or Ncontrol), miR-199a inhibitors (or Inhibitor Ncontrol) were transiently transfected into HEK-293T and Panc-1 cells, and the relative Firefly luciferase to Renilla luciferase activity was measured. We found that the miR-199a mimics reduced the luciferase reporter gene activity more significantly when it was regulated by the T allele variant than the C allele of HIF1A 3′UTR in HEK-293T and Panc-1 cells (P<0.01, P<0.01 respectively) (Figure 3C). We also examined the effect of miR-199a inhibitors on the luciferase activity of two UTR constructs reporter plasmids. Results showed that miR-199a inhibitors significantly obliterated the differences of luciferase activity between two reporter plasmids in HEK-293T and Panc-1 cells (P<0.05, P<0.05 respectively) (Figure 3D). These results demonstrate that miR-199a could suppress expression of the luciferase gene by targeting the 3′UTR of HIF1A gene. This suppressing effect of miR-199a was weakened when the nucleotide at the rs2057482 locus was changed from the ancestral T to the variant C. We therefore propose that C/T variants of rs2057482 could lead to differential regulation of the HIF-1α mRNA by miR-199a and therefore affect the expression of HIF-1α.

## DISCUSSION

Here, we demonstrate, for the first time, that the constitutive expression of HIF-1α in PDAC is regulated by SNPs of HIF1A gene. Overexpression of HIF-1α has been found in many types of human cancers and metastasis [42]. In addition to an induced expression by the hypoxic tumor microenvironment, the precise mechanism of an increased constitutive expression of HIF-1α is still elusive. We found that the CC genotype in the HIF1A rs2057482 is associated with an increased constitutive expression of HIF-1α mRNA/protein, PDAC risk, and adverse clinical outcomes.

There is emerging evidence that genetic alterations located in the 3′UTR of miRNA target genes might affect miRNA-mediated gene regulation and are thus associated with individual susceptibility to cancer development [43]. A SNP in the let-7 miRNA binding site in the 3′UTR of KRAS is associated with risk of lung cancer development [44] and HIF1A c.*191T>C SNP in the 3′ UTR of the gene is significantly correlated with rectal cancer risk [45]. SNP rs2057482 in HIF1A gene is significantly associated with clinical outcomes of Chinese liver cancer patients after surgery [32]. On the basis of this postulation, we hypothesized that the SNP located near the microRNA-199a binding site in the 3′UTR of HIF-1α deregulated in PDAC might be associated with susceptibility to PDAC and poor clinical outcomes.

To assess whether this HIF1A SNPs could confer in PDAC risk, we first conducted a case-control population study and observed that the variant genotypes of HIF1A rs2057482 located 8bp downstream of the binding site of miR-199a confers an increased risk of development of PDAC in this large cohort of Chinese population. The frequency of distribution of HIF1A SNP CC genotype is significantly correlated with tumor size and lymph node metastasis.

HIF-1α expression in PDAC tissue is highly heterogeneous. The CC genotype of SNP HIF1A is correlated with significantly increased HIF-1α mRNA and protein expression but not miR-199a. This suggests that increased expression of HIF-1α is caused by SNPs in HIF1A gene but not the levels of miR-199a. Importantly, both CC genotype of SNP HIF1A and higher HIF-1α protein expression are associated significant reduced OS in patients with PDAC. This association is applicable to PDAC patients after surgical resection and advanced patients after chemotherapy. Using multivariate Cox regression analysis, we found that both the CC genotype of SNP HIF1A and increased HIF-1α expression are significantly associated with survival of PDAC patients with higher TNM staging, differentiation grade, and increased levels of CA19-9. This suggests that both the type of HIF1A SNPs and the levels of HIF-1α expression can be used as biomarkers for predicting clinical outcomes.

To reveal the potential genetic and molecular mechanism of epidemiological observation, we conducted bioinformatic analysis. The MFE of wild miR-199a binding to HIF-1α was 61.9 kcal/mol, while that of mutant form was −59.3 kcal/mol, indicating a lower affinity of variant miR-199a to the binding sites in HIF1A 3′UTR. Thus, we performed a number of wet-laboratory experiments using reporter gene assays and validated that miR-199a indeed targets the predicted region in the HIF1A 3′UTR and that miR-199a represses the T allele more than the C allele. These biochemical findings suggest that miR-199a is a post-transcriptional regulator of HIF-1α expression, supported by the rs2057482 SNP is the risk allele for the development of PDAC by the case-control analysis. We propose that the association between risk of developing PDAC and the rs2057482 SNP in the 3′UTR of the oncogene HIF1A may be due to deregulated control of miR-199a on HIF-1α expression.

This hypothesis has been validated by multiple studies that found that SNPs located within or near the target sites of miRNAs could influence the functions of miRNAs and were associated with human cancers [32, 43, 45]. In this study, we demonstrate that the genetic variants of rs2057482, a SNP located within the 3′ UTR of HIF1A, can weaken the suppression of HIF1A by miR-199a and is associated with the risk of pancreatic cancer and shorter survival of patients with PDAC.

It should be noted that even though in this study we have proven that genetic variants of rs2057482 could influence the regulation of HIF1A by miR-199a, we cannot exclude the possibility that they can also be interfered by other regulatory mechanisms. The first possibility is that genetic variants of rs2057482 can interfere the functions of other miRNAs besides miR-199a, especially considering that there are target sites for several other miRNAs near the binding site of miR-199a according to the target prediction results. This possibility is also demonstrated in the luciferase assays, in which the luciferase activity of both rs2057482-C and rs2057482-T were significantly lower than those of the mutant vector even when no synthesized miR-199a was transfected, and the concentration of endogenous miR-199a is relatively low in HEK-293T cells. A second possible mechanism is that the T/C variants of rs2057482 might alter the functional motif of other regulatory factors apart from miRNAs, even though there is currently insufficient evidence for this possibility. In addition, even though we have demonstrated that T/C variants of rs2057482 can lead to differential suppression of HIF-1α mRNA by miR-199a, we are still unclear about the precise mechanism of how these genetic variants influence the function of miR-199a. Thus, more studies are warranted to elucidate these questions.

**Figure 4:**
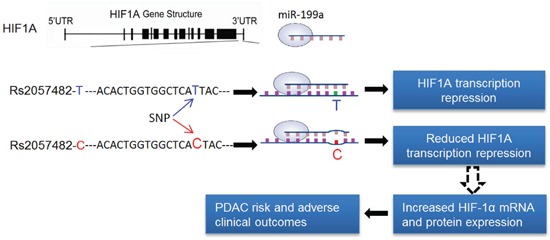
The role of miR-199a in the regulation of HIF1A transcription The variant rs2057482 is a change of C-to-T located at 8bp downstream of the binding site of miR-199a in the 3 UTR of the HIF1A gene. MiR-199a is processed in pancreatic ductal epithelial cells and binds to the 3′UTR of the HIF1A gene. The rs2057482 C allele affects the miR-199a binding site at HIF1A 3′UTR, which damages the binding ability of miR-199a and results in the reduced degradation of HIF-1αmRNA and subsequently increases its translation, which may enhances PDAC risk and the development of PDAC.

In summary, we demonstrate that the constitutive increased expression of HIF-1α is regulated by SNP rs2057482 within the 3′ UTR of HIF1A gene, suggesting that increased HIF-1α expression is not only induced by hypoxic microenvironment but also genetic modification in the gene. Most importantly, both the CC genotype of SNP HIF1A and overexpression of HIF-1α are significantly associated with poor clinical outcomes of patients with PDAC (Figure 4). We therefore propose that HIF1A gene or HIF-1α protein is a potential target for the treatment of PDAC and the biomarker for predicting clinical outcomes.

## MATERIALS AND METHODS

### PDAC patients and control subjects

PDAC samples used for this study were from patients previously treated in the Tianjin Medical University Cancer Institute and Hospital (TMUCIH) from February 2011 to February 2015 (n=410). 490 out of 760 healthy, cancer-free native men and women were randomly sampled from who visited TMUCIH for physical check-up, were recruited as controls. The controls were genetically unrelated to the patients and were frequency (sex and age) matched to the patients. All participants enrolled in the study were of Chinese Han ethnicity. Each participant gave us informed consent prior to participation. Informed consent was obtained from all participants, epidemiology questionnaires were administered and blood and/or tissue were obtained. The epidemiology questionnaire included personal history, smoking history, pancreatitis history, type II diabetes history. All PDAC tumors were confirmed by cytology. Tumor staging was based on the T/N/M system. The study was proved by the local ethics committee.

### Computational analysis

The miRBase, the Gene and the dbSNP databases of the National Center for Biotechnology Information (NCBI), and the International HapMap Project database were first employed to identify SNPs located within the human HIF1A and human miR-199a genes that had potential functional effect. The coding sequence (CDS), promoter region (defined as the 2 Kb sequence upstream of the transcriptional start site of HIF1A), and 3′UTR of HIF1A were located using Gene database of NCBI. The genes of human miR-199a were determined through data curated by miRBase and confirmed through Gene database of NCBI. TargetScan Human 5.1, PicTar, and MicroCosm Targets Version 5 were employed to predict the target sites of miRNAs. RNAfold software (http://rna.tbi.univie.ac.at/) was used to calculate the secondary structure of RNA based on minimum free energy (MFE).

### Genomic DNA extraction, amplification and genotyping

Peripheral blood samples were obtained from each participant. Mononuclear cells were isolated by Ficoll-Paque separation. Genomic DNA was extracted using the Axygen DNA isolation kit (Axygen). The rs2057482 in the gene of HIF-1α were detected by polymerase chain reaction (PCR) using a Mastercycler^®^gradient thermal cycler (Eppendorf^®^, Germany). The PCR mix (25 μl) contained genomic DNA (500 ng), MgCl_2_ (2.5 mM), dNTP (100 mM), Taq polymerase (1.0 U/ml) and primers (forward 5′-TAC AAG GCA GCA GAA ACC-3′ and reverse 5′-TAA ACT CCC TAG CCA AAA-3′, 50 pmol/ml for each). After a 10 min denaturation at 94°C, the mix underwent 30 cycles of denaturation (94°C, 45 sec), annealing (60°C, 45 sec) and extension (72°C, 45 sec). PCR products were verified by agarose gel electrophoresis. DNA fragments were sequenced in Sangon Biological Engineering Technology and Services (Shanghai, China) using 5′-GTG GAT AGT GAT ATG GTC AAT G-3′ as the primer. Sequence analysis was performed using the program of BioEdit. When C/T genotypes were found, the analysis was repeated to double-check the results.

### Cell lines and cell culture

The human pancreatic cancer Panc-1 cell line and the human embryonic kidney HEK-293T cell line were cultured in Dulbecco's modified Eagle's medium supplemented with 10% fetal bovine serum at 37°C in a humidified atmosphere with 5% CO_2_.

### RNA extraction

Total RNAs of 60 PDAC tissue samples (40 with CC genotypes and 20 with CT/TT genotypes of rs2057482) were isolated using Trizol reagent (Invitrogen). A 2 mm^3^frozen tissue sample was homogenized in 1 ml of Trizol reagent. The extraction was performed according to the Invitrogen protocol. RNA concentrations were measured using Nano Drop ND-1000 spectrophotometer, and RNA quality was assessed by running the RNA on 1.0% agarose gels.

### Real-time RT-PCR analysis of miRNA and mRNA

Total RNA was extracted using Trizol, and reverse-transcribed into cDNA with the Reverse Transcriptase M-MLV (TaKaRa). Real time PCR was performed using a SYBR Premix Ex Taq™ kit (TaKaRa) on the ABI PRISM 7500 sequence detection system (Applied Biosystems, Foster City, USA). In each assay, 1 μl of cDNA was used for amplification. The reactions were incubated in a 96-well optical plate at 95°C for 2 min, followed by 40 cycles of 95°C for 15 s and 60°C for 20 s. PCR primers used were as follows: HIF-1α F, 5′-GCA AGC CCT GAA AGC G-3′ and R, 5′-GGC TGT CCG ACT TTG A-3′; β-actin F, 5′-CAG AGC AAG AGA GGC ATC C-3′ and R, 5′-CTG GGG TGT TGA AGG TCT C-3′. For analysis of miRNA expression by qRT-PCR, reverse transcription and PCR were carried out using Bulge-Loop TM miRNA qPCR Primer Set for hsa-miR-199a (RiboBio, MQP-0101), and U6 snRNA (RiboBio, MQP-0201) according to the manufacturer's instructions. Expression of HIF-1α, relative to β-actin and miR-199a, relative to U6, was determined using the 2^−ΔΔCT^method. All qRT-PCRs were performed in triplicate, and the data are presented as means ± standard errors of the means (SEM).

### Vectors construct and site-directed mutagenesis

A 1340-bp fragment of the 3′UTR of human HIF1A gene (accession number: NM181054) centering rs2057482C and the predicted complementary site of miR-199a (ACACTGG) was subcloned into the pmiR-RB-REPORT™ reporter plasmid (RiboBio Co Ltd, China) using the Xhol and NotI restriction sites located 3′ to the Renilla luciferase translational stop codon. A mutant plasmid with T allele at the site of rs2057482 was constructed using site-directed mutagenesis primers based on rs2057482C HIF1A 3′UTR plasmid. A mutant HIF1A 3′-UTR plasmid for the predicted complementary site of miR-199a (TCTCAGC) with C allele at the site of rs2057482 was constructed using a site directed mutagenesis kit (Stratagene, USA). All vectors were verified by direct sequencing.

### Luciferase reporter assay

To conduct the luciferase reporter assay, HEK-293T cells and Panc-1 cells were cultured in 24-well plates at 1×10^5^cells/well. After an overnight incubation, each well was treated with a transfection mixture consisting of 500 μl of Opti-MEM (Invitrogen, Carlsbad, CA, USA), 100 ng of pmiR-RB-REPORT™ reporter plasmid (rs2057482C, rs2057482T, or Mutant), and 100 nmol of synthesized miRNA (miR-199a mimics, Mimic Ncontrol, miR-199a inhibitors, Inhibitor Ncontrol) (RiboBio Co Ltd, China) using Lipofectamine 2000 according to the protocol (Invitrogen, Carlsbad, CA). After 5 hours incubation, the media were replaced by 500 μl of serum-containing medium. After 48 hours, the Renilla and firefly luciferase activities were measured by the Dual-Luciferase Reporter Assay (Promega, USA) on a Veritas Microplate Luminometer (Turner BioSystems, USA) according to the manufacturers' protocol. Each experiment was performed at least three times in triplicate. The luciferase score was calculated by normalizing the luciferase signal of Renilla to that of firefly. Fold change (relative luciferase activity) was reported by setting the scores of the control groups as one and normalizing the scores in other groups.

### Immunohistochemistry

PDAC specimens were embedded in paraffin, sectioned (4-μm thick) and deparaffinized. All sections were treated with 5 mM citrate buffer for antigen retrieval and with 3% H_2_O_2_ for inactivation of endogenous peroxidase. Next, sections were incubated with a blocking buffer for 30 min and with a HIF-1α antiserum overnight (sc-10790, Santa Cruz, dilution: 1:200, at 4°C). Afterwards, the sections were incubated for 30 min with a secondary antibody (Maixin Corporation, China). Diaminobenzidine was used as a chromogen substrate. All sections were counterstained with hematoxylin. In a light microscope, 4 random fields (200X) were examined [46]. In each tumor sample, the score of HIF-1α-positive cell frequency was multiplied with that of staining intensity. Staining intensity was scored 0 (negative), 1 (low), 2 (medium), and 3 (high). Staining extent was scored 0 (0% stained), 1 (1%–25% stained), 2 (26%–50% stained), and 3 (51%–100% stained). The final score was determined by multiplying the intensity scores with staining extent and ranged from 0 to 9. Final scores (intensity score × percentage score) less than 3 were considered as low staining (+), 4–6 were medium staining (++) and >6 were high staining (+++).

### Statistical analysis

Categorical data are described as frequency of the subjects. Continuous data are expressed as mean ± SD. Chi-square test or Fisher's exact test was used for comparing categorical variables. The Hardy-Weinberg equilibrium was performed by χ^2^test. The Student *t* test, Oneway ANOVA test were used for comparing continuous variables. The association between genotypes or alleles with the risk of PDAC was determined using logistic regression (OR and 95% confidence interval, Cl). The association between HIF1A genotypes and HIF-1α expression was calculated by Spearman's rank correlation test. The log rank test and Kaplan–Meier survival curves were used to analyze the association between the studied genotypes or HIF-1α expression on patient's overall survival (OS) and relapse-free survival (RFS) to obtain *P* values, hazard ration (HR) and 95% CI. Univariate analysis was performed first, including one factor a time to predict their effects on the clinical outcome. Multivariate analysis was performed using a Cox proportional hazards model to assess the independent effect of prognostic variables on the outcome. Relevant factors were considered simultaneously through the use of both forward and backward stepwise models. All statistical calculations were performed using the SPSS (version 22.0) and GraphPad Prism (version 5.03). All *P*-values less than 0.05 were considered statistically significant. *, **, and *** indicate *P*-value < 0.05, 0.01, and 0.001, respectively [46].

## SUPPLEMENTARY FIGURES AND TABLES


