# Supplementary material for: Single nucleotide polymorphism in the microRNA-199a binding site of HIF1A gene is associated with pancreatic ductal adenocarcinoma risk and worse clinical outcomes
Source: Oncotarget. 2016 Feb 8;7(12):13717–29. doi: 10.18632/oncotarget.7263 (PMC4924673; doi:10.18632/oncotarget.7263)
Supplement: Supplementary file 1 [file oncotarget-07-13717-s001.pdf]

SUPPLEMENTARY FIGURES AND TABLES

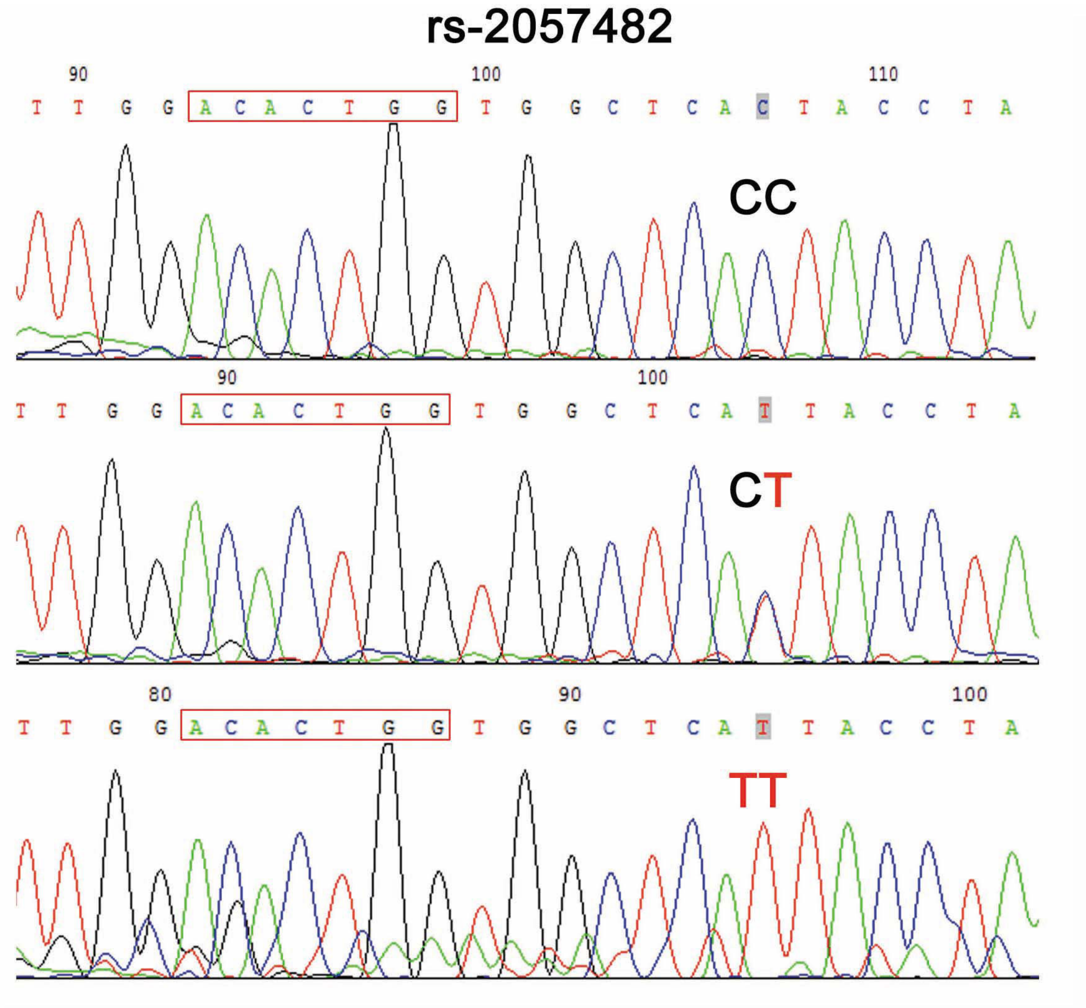

Supplementary Figure 1: Direct sequencing results for the HIF1A gene SNP (rs2057482 CC, CT and TT).

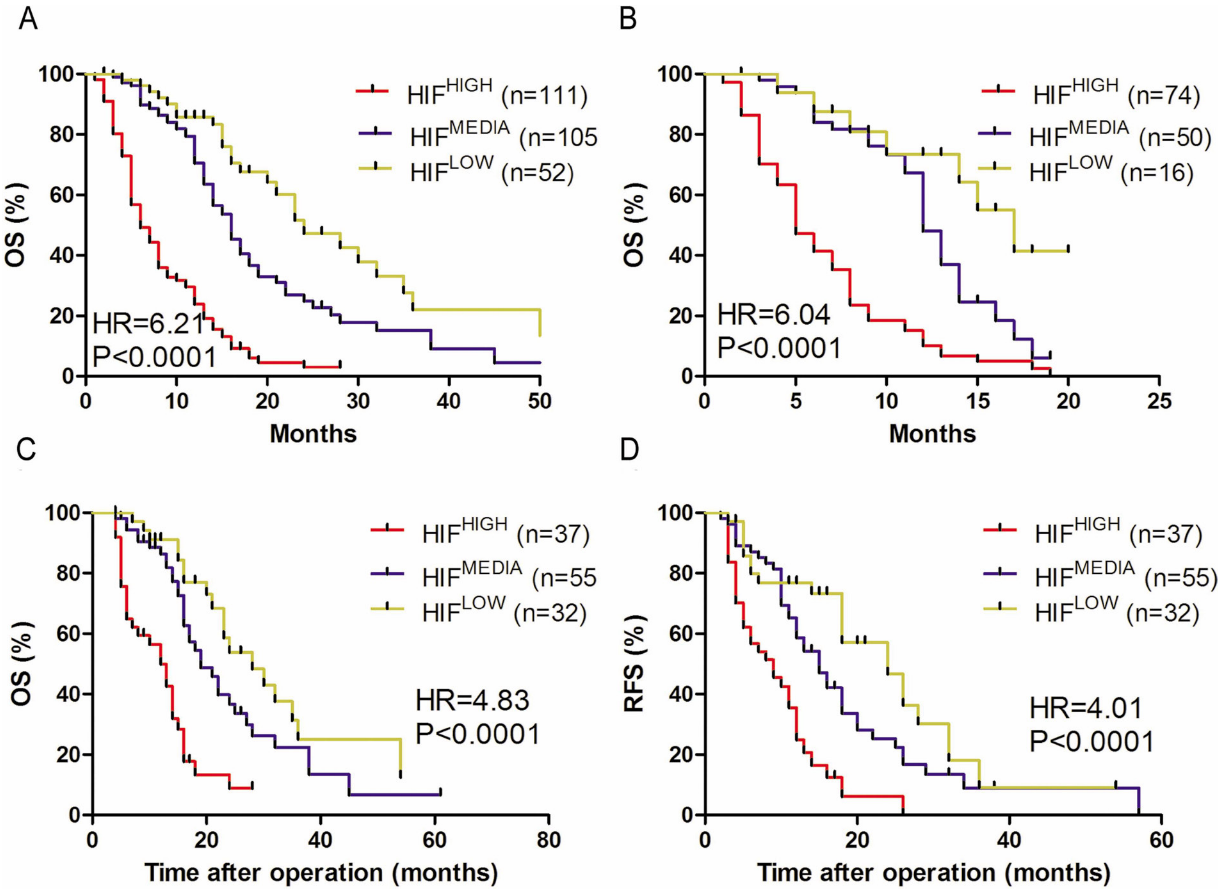

Supplementary Figure 2: The association between HIF-1α protein expression and survival rate in patients with PDAC. The levels of protein expression were defined as low, media and high according to histo-pathological scores. **A.** Kaplan–Meier survival curves of overall survival (OS) of 269 PDAC patients; **B.** Kaplan–Meier curves of OS for 141 advanced PDAC patients; **C, D.** OS (C) and relapse-free survival (RFS) (D) of 128 PDAC patients who undergoing surgical resection. Data were analyzed by the log-rank test and the Kaplan–Meier curves were generated by using GraphPad Prism software. The P value and the hazard ratio in each graph present data of higher HIF-1α verses median HIF-1α expression.

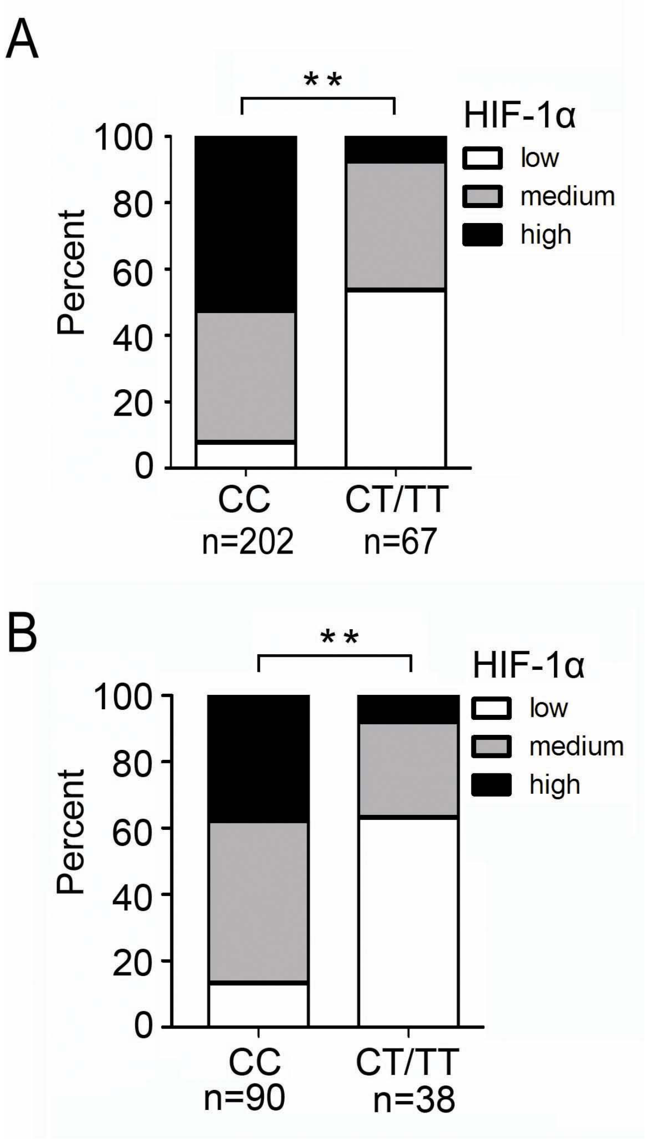

Supplementary Figure 3: The frequency distribution of HIF-1α expression between CC and CT/TT genotype PDAC groups.

Supplementary Table 1: Multivariate correlation analysis

			TNM	T	N	Grade	SNP199_2	HIF1	CA199_2	age
Spearman's rho	TNM	Correlation Coefficient	1.000	.290**	.483**	.085	-.079	.318**	.052	.006
		Sig. (2-tailed)		.000	.000	.165	.195	.000	.396	.916
		N	269	269	269	269	269	268	269	269
	Tumor size	Correlation Coefficient	.290**	1.000	.337**	.028	-.185**	.196**	-.010	.144*
		Sig. (2-tailed)	.000		.000	.650	.002	.001	.869	.018
		N	269	269	269	269	269	268	269	269
	LN metastasis	Correlation Coefficient	.483**	.337**	1.000	.118	-.140*	.186**	.037	-.009
		Sig. (2-tailed)	.000	.000		.052	.022	.002	.545	.888
		N	269	269	269	269	269	268	269	269
	Grade	Correlation Coefficient	.085	.028	.118	1.000	-.115	.273**	.029	.014
		Sig. (2-tailed)	.165	.650	.052		.059	.000	.635	.818
		N	269	269	269	269	269	268	269	269
	SNP	Correlation Coefficient	-.079	-.185**	-.140*	-.115	1.000	-.511**	.015	.003
		Sig. (2-tailed)	.195	.002	.022	.059		.000	.808	.957
		N	269	269	269	269	269	268	269	269
	HIF1a protein	Correlation Coefficient	.318**	.196**	.186**	.273**	-.511**	1.000	.242**	.061
		Sig. (2-tailed)	.000	.001	.002	.000	.000		.000	.318
		N	268	268	268	268	268	268	268	268
	CA199	Correlation Coefficient	.052	-.010	.037	.029	.015	.242**	1.000	-.068
		Sig. (2-tailed)	.396	.869	.545	.635	.808	.000		.264
		N	269	269	269	269	269	268	269	269
	age	Correlation Coefficient	.006	.144*	-.009	.014	.003	.061	-.068	1.000
		Sig. (2-tailed)	.916	.018	.888	.818	.957	.318	.264	
		N	269	269	269	269	269	268	269	269

**. Correlation is significant at the 0.01 level (2-tailed).
*. Correlation is significant at the 0.05 level (2-tailed).

Supplementary Table 2

A: HIF-1α expression is stratified by HIF1A SNPs

	B	SE	Wald	df	Sig.	Exp(B)	95.0% CI for Exp(B)	
							Lower	Upper
HIF1			54.756	2	.000			
HIF1(1)	.372	.259	2.064	1	.151	1.450	.873	2.409
HIF1(2)	1.501	.265	32.101	1	.000	4.487	2.669	7.542

B: HIF-1α expression is adjusted by HIF1A SNPs

	B	SE	Wald	df	Sig.	Exp(B)	95.0% CI for Exp(B)	
							Lower	Upper
HIF1			59.966	2	.000			
HIF1(1)	.332	.253	1.730	1	.188	1.394	.850	2.289
HIF1(2)	1.516	.258	34.574	1	.000	4.553	2.747	7.547
SNP199_2	.652	.225	8.407	1	.004	1.919	1.235	2.982
